# Modulation of Homology-Directed Repair in T98G Glioblastoma Cells Due to Interactions between Wildtype p53, Rad51 and HCMV IE1-72

**DOI:** 10.3390/v6030968

**Published:** 2014-02-26

**Authors:** Amit S. Kulkarni, Elizabeth A. Fortunato

**Affiliations:** 1 Tumorvirologie (F010), Deutsches Krebsforschungszentrum, Im Neuenheimer Feld 242, 69120, Heidelberg, Germany; E-Mail: amitkulkar@gmail.com; 2 Department of Biological Sciences and the Center for Reproductive Biology, University of Idaho, 875 Perimeter Drive, Mailstop 3051, Moscow, ID 83844, USA

**Keywords:** human cytomegalovirus, homology directed repair, p53, IE72, Rad51

## Abstract

Human cytomegalovirus (HCMV) is a ubiquitous pathogen capable of causing life threatening consequences in neonates and immune-compromised individuals. HCMV inflicts site-specific double strand breaks (DSBs) in the cellular genome. DNA damage infliction raises the corollary question of virus modulation of DNA repair. We recently reported HDR was stimulated in wt human foreskin fibroblasts (HFFs) during fully permissive infection or expression of the HCMV protein IE1-72 (IE72). These studies have been extended into semi-permissive T98G glioblastoma cells. T98Gs encode a mutant p53, which may contribute to their high baseline rate of HDR. We fully expected HCMV infection to increase HDR in T98Gs, similar to its effects in HFFs. Surprisingly in T98Gs HCMV infection, or sole expression of IE72, decreased HDR by two-fold. Transient expression of wt p53 in T98Gs also reduced HDR by two-fold. Dual transient expression of wt p53 and IE72 restored high baseline HDR levels. GST pulldown experiments revealed that both IE72 and wt p53 bound the important HDR protein, Rad51. We conclude that the expression of certain HCMV proteins can modulate HDR in an infected cell, dependent upon p53 status. We propose a model of the protein interactions explaining this behavior.

## 1. Introduction

Human cytomegalovirus (HCMV) is a member of the β-herpesvirus family and is endemic in the human population. HCMV can cause congenital birth defects, primarily of neurological origin [1,2,3]. It also causes severe systemic disease following reactivation in immunosuppressed individuals [4]. The virus’ role in oncomodulation of cancers has also recently been the subject of intensive investigation (for review see [5,6]). 

One of the direct effects of HCMV infection in human foreskin fibroblasts (HFFs), is the induction of two site-specific breaks at chromosome 1q42 and 1q23.3 in the host cellular DNA [7,8]. The physiological significance of HCMV-induced specific breaks or whether they can be repaired is unknown. However, the proximity of the 1q23.3 breakpoint to two hearing impairment (HI) loci, DFNA49 and DFNA7, and in the vicinity of the myelin protein zero (MPZ) gene [9], prompts speculation on this break’s correlation with the hearing loss observed in congenitally infected infants.

Mammalian cells are exposed to both intrinsic (*i.e.*, reactive oxygen species) and extrinsic (*i.e.*, genotoxic chemicals) agents capable of inflicting cellular DNA damage. DNA damage triggers a cascade of complex protein signaling pathways. This combination of pathways constitutes the DNA damage response (DDR). Depending on the type and extent of DNA damage different repair pathways are activated. In the case of double-stranded breaks (DSBs), two repair pathways are principally activated (as reviewed in [10,11]). One is the error free homology-directed repair (HDR) pathway, which commonly involves strand invasion of a sister chromatid. The other pathway utilizes the more error-prone non-homologous end-joining (NHEJ) or single-strand annealing mechanisms.

We recently reported interactions between HCMV and HDR in permissive HFFs [12]. HCMV infection caused a two-fold increase in HDR of an I-*Sce*I induced site-specific DNA DSB, from a baseline level of 4% to a stimulated level of 8%. Expression of the HCMV IE72 protein on its own produced comparable results. Here the same DSB repair assay has found that HCMV regulates HDR in the semi-permissive T98G cells in a surprising and counterintuitive manner, hinging on the p53 status of these cells. A model to explain the interaction of HCMV IE72 with p53 and the HDR machinery is proposed. 

## 2. Results

### 2.1. Introduction of the pDRGFP Substrate into T98G Cells to Assess HDR

**Figure 1:**
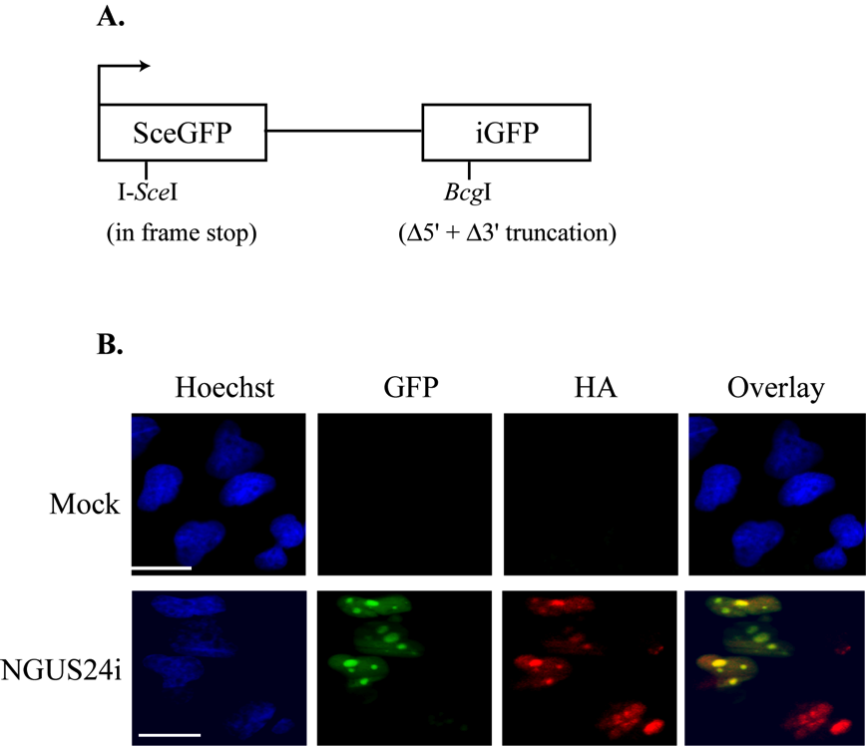
Efficient expression of I-*Sce*I from an Adenoviral vector based system in T98Gs with an integrated pDRGFP substrate. (**A**) Schematic diagram of reporter plasmid pDRGFP as described in [12]. (**B**) Representative IF staining of Ad-infected Clone 10. Cells were seeded onto glass coverslips and infected with Ad expressing I-*Sce*I (NGUS24i). Coverslips were harvested at 72 h post infection. GFP^+^ cells were scored as HDR competent. Cells were stained with HA-specific Ab to detect HA-I-*Sce*I expressed from NGUS24i. Scale bar = 5 µm for all panels and all figures.

Our earlier study assessed whether HCMV infection affected HDR of an integrated substrate at I-*Sce*I-induced DSBs in fully permissive HFFs. This work found that HDR, as measured by an increase in GFP^+^ cells, was stimulated in HFFs during HCMV infection and, further, that expression of the HCMV protein IE72 on its own also stimulated HDR [12]. The assay utilized a non-functional GFP reporter substrate susceptible to DSBs upon introduction of I-*Sce*I [13,14] (see Figure 1A for diagram of the substrate). If the DSB was successfully repaired by HDR, functional GFP was reconstituted. HDR was scored by microscopic analysis for cellular GFP^+^. These fibroblast experiments have now been extended into the semi-permissive cell line T98G. Three different stable clones of T98Gs with an integrated pDRGFP substrate were generated. The results obtained using all clones were consistent, therefore here we report only representative studies carried out in Clone 10. 

Clone 10 was synchronized in G_0_ and then infected with an Adenovirus (Ad) expressing the I-*Sce*I endonuclease (NGUS24i), or an Ad control virus (dL70-3) [15,16] at various MOIs (5, 10, 25, 50, 100, and 200) or were mock-infected. Cells were analyzed for efficiency of I-*Sce*I endonuclease production after NGUS24i infection. The I-*Sce*I endonuclease in this virus is tagged with hemagglutinin (HA), permitting detection of protein expression using an anti-HA Ab by IF. 100% of the cells infected with NGUS24i displayed HA-positivity and no staining was observed in mock-infected cells (Figure 1B) or dL70-3-infected cells (data not shown).

NGUS24i infection produced GFP^+^ cells. This indicated I-*Sce*I-induced site-specific DNA breaks had been successfully repaired by HDR. In our hands NGUS24i infection effects were greatest using an MOI of 200 and continuous incubation for 72 h prior to harvest. Clone 10 was also examined for spontaneous HDR events. Figure 1B displays representative experiments depicting the absence of any GFP^+^ cells in mock-infected Clone 10. These results demonstrated that the Ad-based enzyme delivery system in conjunction with the integrated pDRGFP substrate worked as well in the T98G cells as had been previously observed in the HFFs [12].

### 2.2. HDR at I-SceI Induced DSBs Was Downregulated in HCMV-Infected T98G Cells

**Figure 2:**
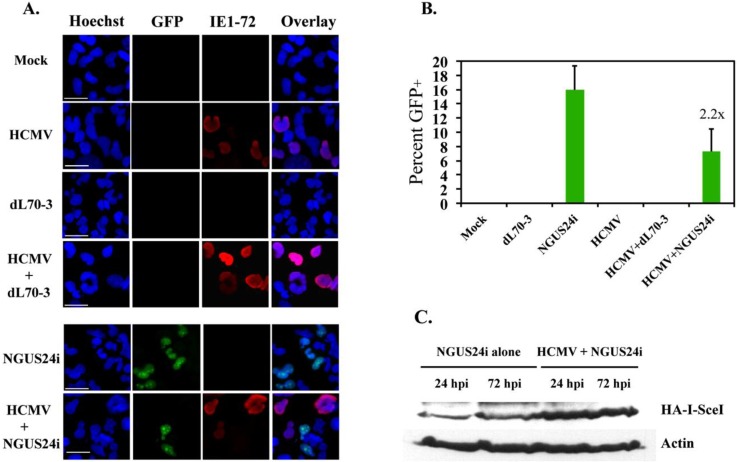
HCMV infection decreased HDR in T98Gs in the presence of stably integrated pDRGFP substrate. Cells were seeded onto glass coverslips and infected with HCMV or Ad individually or dually with HCMV and Ad in succession. Coverslips were harvested at 72 h post Ad superinfection with either I-*Sce*I expressing (NGUS24i) or control (dL70-3) virus. GFP^+^ cells were scored as HDR competent. Clone 10 was stained by IF for the presence of IE72. (**A**) Representative IF staining for GFP^+^ + IE72^+^ cells in Clone 10. (**B**) Percentage of GFP^+^ cells in Clone 10 after infection. (**C**) Representative protein profile for steady state levels of HA-tagged I-*Sce*I in Clone 10 infected with NGUS24i alone or dually infected with HCMV and NGUS24i. Actin was used as a loading control. For IF studies at least 300 cells were scored for each type of infection. Values on top of the bar indicate fold decrease in percent of GFP^+^ cells in dually infected *versus* NGUS24i alone experiments. Error bars represent one SD.

Previously we showed that in a semi-permissive infection of T98G cells, although all cells take up virus, only a modest percentage (~30%–40%) of cells express IE72 antigen (Ag) in contrast to 100% Ag^+^ cells in a fully permissive infection [17]. Clone 10 was G_o_ synchronized, released from synchronization and then mock-infected or HCMV-infected at an MOI of 10 for 48 h. These cells were subsequently superinfected with NGUS24i or dL70-3 and harvested at 72 h post Ad infection. Cells were stained for the expression of HCMV IE72 by IF to monitor initiation and progression of HCMV infection. Approximately 30% of cells were IE72^+^ at 120 h post infection (hpi), which agreed with previous experiments [17]. Cells were also scored for GFP^+^. Representative IF images for these infections are shown in Figure 2A. 

T98Gs displayed a very high baseline rate of HDR after DSB induction with I-*Sce*I. Ad NGUS24i infection resulted in ~16% GFP^+^ cells (as compared to 4% in HFFs) [12]. No GFP^+^ cells were observed in either dL70-3-infected or mock-infected cells. An average of ~7% GFP^+^ cells were detected in HCMV-infected cells subsequently superinfected with NGUS24i to induce DSBs, an ~2 fold decrease (Figure 2B). Although not quite reaching a degree of statistical significance, this decrease was consistently reproducible (fold changes of 2.7 and 1.9 in two experiments). Downregulation of HDR due to HCMV infection was not associated with changes in steady state levels of I-*Sce*I expression. Immunoblot analysis found no decrease in I-*Sce*I expression in HCMV-infected T98Gs compared to mock-infected T98Gs (Figure 2C). Actually, somewhat the reverse was found with slightly higher levels of I-*Sce*I observed in lysates derived from an equivalent number of virus-infected cells. Thus lower expression of the I-*Sce*I enzyme was not the cause of the somewhat paradoxical decrease in HDR.

### 2.3. Transient Expression of IE72 Alone Was Sufficient to Downregulate HDR in T98Gs

A number of studies have suggested that IE72 plays a vital role in fine tuning the host cell, including activating early gene expression needed for viral replication ([18] and as reviewed in [19]). We had previously shown that expression of IE72 alone was sufficient to enhance HDR in HFFs [12]. Having found that full HCMV infection reduced HDR in T98Gs, we repeated the IE72 expression assays in these cells. Parallel experiments to those above were performed in Clone 10 nucleofected with vector-alone (pSG5) or an IE72-expressing construct (pSGIE72). These cells were then Ad infected. Cells were subsequently scored for IE72 and GFP positivity. Approximately 90% cells were IE72^+^ (data not shown). 

**Figure 3:**
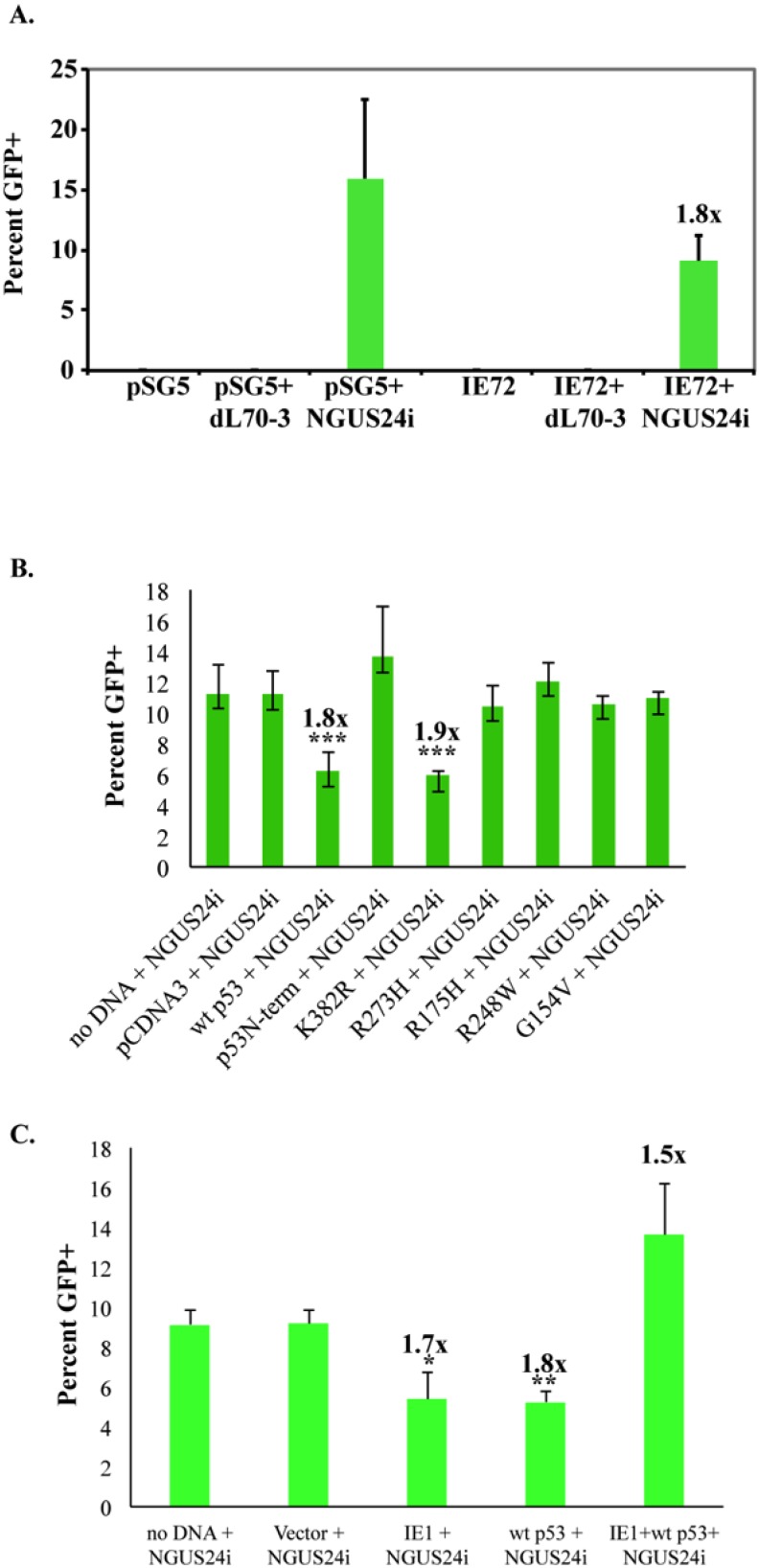
Nucleofection with IE72 or wt p53 alone decreased HDR, whereas co-expression did not alter high level HDR in T98Gs. Cells were nucleofected with the indicated plasmids and then infected with either I-*Sce*I expressing (NGUS24i) or control (dL70-3) Ad. Coverslips were harvested at 40 h post Ad infection. GFP^+^ cells were scored as HDR competent. (**A**) Percentage of GFP^+^ in Clone 10 after nucleofection of IE72 or pSG5 control. (**B**) Percentage of GFP^+^ in Clone 10 nucleofected with either wt p53 or p53 mutants (K382R, N-term, R273H, R175H, R248W, G154V). (**C**) Percentage of GFP^+^ in Clone 10 nucleofected with wt p53 and/or IE72. At least 300 cells were scored for each type of infection. Error bars represent one SD. Values on top of the bars indicate fold decrease (or increase for the dual IE72/wt p53 nucleofection) in percent of GFP^+^ cells. *, ** and *** indicate increasing levels of statistical significance (see text for details). Error bars represent one SD.

No GFP^+^ cells were observed in vector alone (pSG5), pSG5 + dL70-3-infected (control Ad), IE72 alone or IE72 + dL70-3-infected cells. In close agreement with the previous results an average of ~16 % GFP^+^ cells were detected in cells nucleofected with pSG5 and NGUS24i-infected (I-*Sce*I expressing Ad). In cells nucleofected with the IE72 construct and NGUS24i-infected, an average of 9% GFP^+^ cells were scored, a 1.8 fold decrease (Figure 3A). This decrease was approximately equivalent to the reduction in GFP^+^ of HCMV-infected cells. Again, although this decrease was not statistically significant, it was consistently reproducible (fold changes of 1.9 and 1.5 in two experiments). Therefore, as was seen in HFFs, transient expression of IE72 alone largely recapitulated the HCMV-infection results, however, with the same perplexing downregulation of HDR in T98Gs.

### 2.4. Expression of wt p53 in T98Gs Caused the Same Decrease in HDR as IE72

T98Gs have a mutagenic profile and harbor a mutation in p53 at R273H, in the DNA binding domain [20]. It is widely accepted that wt p53 suppresses HDR (as reviewed in [21]). Was the mutation in p53 responsible for the elevated levels of HDR (~16%, Figure 2B) in uninfected T98Gs compared to HFFs (~4%, [12])? If so, introduction of wt p53 would be expected to decrease HDR levels in uninfected T98G cells and, similarly, p53 DNA binding domain mutants would not be expected to alter HDR levels. Experiments parallel to those discussed for IE72 were performed in Clone 10. It should be noted that although all the constructs used in this study were GFP-tagged, the wt p53 and the mutant p53 genes were expressed from the weak wt p53 promoter and, as we have previously observed [22], no detectable GFP expression above background was observed in control populations of these cells (data not shown). Clone 10 was nucleofected with vector-alone (pCDNA3-T7) or constructs [22] expressing wt p53, a non-acetylatable p53 mutant (K382R), p53 DNA binding domain mutants (R273H, R175H, R248W, G154V), or a p53 N-terminal multi-site phosphorylation mutant and subsequently Ad-infected. Cells were scored for GFP^+^ (Figure 3B).

Immediately evident was that introduction of even low levels of wt p53 into T98Gs resulted in accelerated cell death, with very few cells surviving until 72 h post Ad infection. The infection protocol was modified to overcome this technical problem. Ad infection MOIs were reduced from 200 to 5. An MOI of 5 still delivered I-*Sce*I enzyme to the entire population, as detected by HA Ab staining (data not shown), however the lower MOI reduced baseline DSB-induced HDR events to a more modest, but still prevalent, ~11%. More importantly, the reduced MOI allowed sufficiently high cell survival for a long enough duration to obtain meaningful population counts. Eight hours post‑nucleofection, cells were Ad infected and GFP^+^ was scored at 40 h post Ad infection. Following this modified protocol, control experiments using any of the constructs followed by infection with the dL70-3 control virus showed no GFP^+^ cells (data not shown). Nucleofection with no DNA, or introduction of vector alone (pCDNA3) followed by I-*Sce*I-expressing NGUS24i-infection produced an average ~11% GFP^+^ cells (Figure 3B). Cells nucleofected with wt p53 or the p53 K382R mutant produced an average of ~6% GFP^+^, an ~2 fold decrease. This decrease was consistently reproducible and highly statistically significant for both constructs (*p* value < 0.0001 and 0.0004, respectively). The introduction of the p53 DNA binding domain mutants (R273H, R175H, R248W, G154V), or the p53 *N*-terminal multi-site phosphorylation mutant yielded no change in the percent of GFP^+^ from the vector alone. Thus, introduction of wt p53 into the T98G cells suppressed the high level HDR allowed by the endogenous mutant p53 [20]. 

### 2.5. Co-Expression of IE72 and wt p53 Negated Their Individual Effects on HDR

Surprisingly, in the T98G microenvironment, expression of either IE72 or wt p53 reduced HDR, therefore we tested co-introduction of both proteins. Using the modified protocol described above, once again control experiments using dL70-3 virus produced no GFP^+^ cells (data not shown). We observed that nucleofection with no DNA, or introduction of backbone vector (pCDNA3) followed by I-*Sce*I-expressing NGUS24i produced an average ~9% GFP^+^ cells. Introduction of either IE72 or wt p53 alone reduced GFP^+^ to ~5%, an ~2 fold decrease as expected, both of which were statistically significant (*p* = 0.01 and *p* = 0.0014, respectively). Much to our surprise, dual introduction of IE72 and wt p53 produced an average of 13.6% GFP^+^ cells, an ~1.4 fold increase from the baseline rate of ~9%, an increase that was only marginally statistically significant (*p* = 0.043) (fold changes of 1.3, 1.7, 1.5 in three experiments) (Figure 3C). This suggested that in T98G cells interaction between wt p53 and IE72 negated their individual effects on the HDR machinery.

### 2.6. *In Vitro* Binding Assays Found Both wt p53 and IE72 Bound Rad51

**Figure 4:**
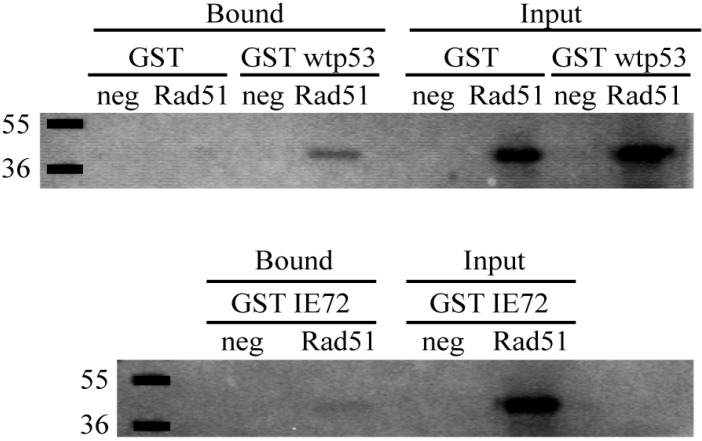
GST pulldown experiments revealed IE72 and wt p53 bound to Rad51. GST proteins were incubated with radiolabeled Rad51 for 1 h as described in experimental details. Input lanes represent 1/10th the total starting reaction. Negative controls are incubations with *in vitro* translation mixture containing an equivalent amount of radioactivity.

**Figure 5:**
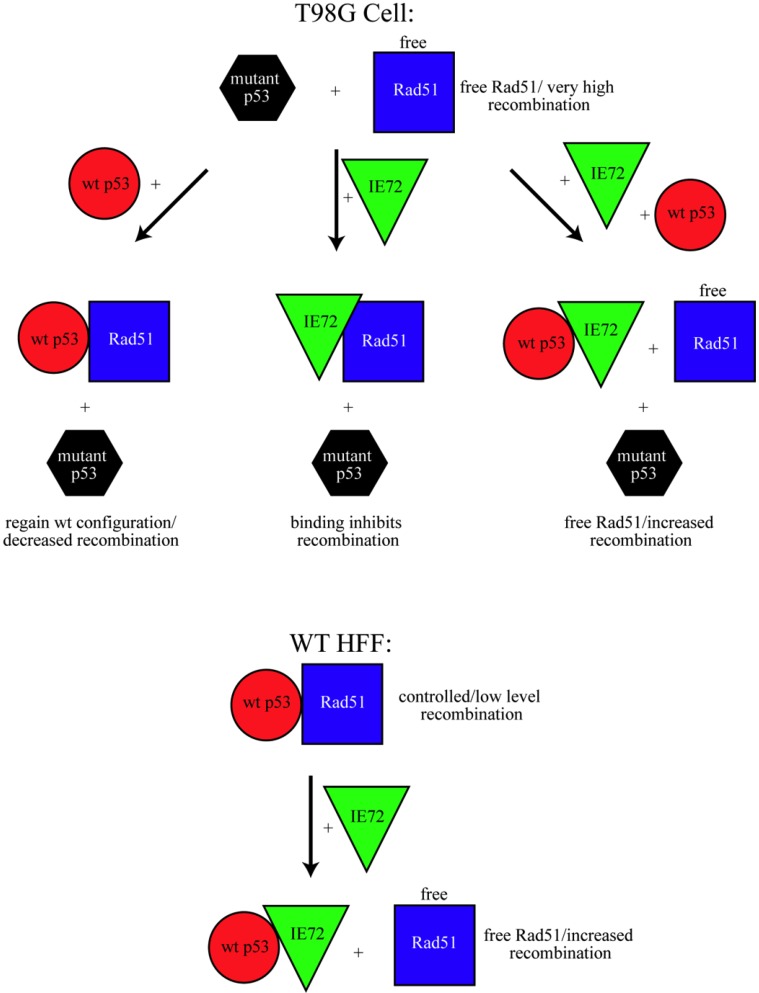
A model for the interactions between wt p53, Rad51 and IE72 in HFFs and T98Gs. Interactions between these three proteins and the ramifications to HDR depend completely on the cellular microenvironment and the presence or absence of wt p53. See text for description.

T98G cells harbor a mutant p53 (R273H mutation) [20]. The mutation abolishes specific DNA binding. The p53 transient expression experiments established that an intact DNA binding domain and phosphorylatable N-terminus were required to decrease HDR in these cells. Previous studies have determined that recombination is controlled, at least in part, by p53 binding to the strand invasion protein Rad51, which modulates Rad51’s function (as reviewed in [21]). An intact DNA binding domain in the p53 protein is required for this interaction [23,24,25]. Further, T98G cells mount a DSB response [20] and express high levels of the DSB repair proteins, including Rad51 [20,26]. Previous work had established that p53 could bind both Rad51 and IE72 [27], and that interaction between p53 and IE72 could negate p53’s normal DNA binding ability through its core region [27]. This information suggested that the effects noted in the above transient expression and co-expression experiments might be the result of IE72 binding either wt p53 or Rad51. *In vitro* mixing experiments of radiolabeled Rad51 and pGEX72, GST wt p53 or pGEX-KG (control GST alone) were performed. As can be seen in Figure 4, both p53 and IE72 were capable of binding Rad51, although IE72 appeared to be slightly less avid for the protein. While not excluding other possible explanations, this binding, in combination with the results from the expression experiments, have prompted us to propose a protein interaction model capable of explaining the observed behaviors (see Figure 5 below). This model may have significant bearing on questions entirely unrelated to HCMV-infection in the semi‑permissive cell type T98G.

## 3. Experimental Section

### 3.1. Cells and Virus Growth

T98G glioblastoma cells and clones expressing pDRGFP were propagated in Earle’s minimal essential media (MEM) supplemented with 10% heat-inactivated fetal bovine serum (FBS), L‑glutamine (2 mM), penicillin (200 U/mL), streptomycin (200 mg/mL), and amphotericin B (1.5 mg/mL). Cells were grown in incubators maintained at 37 °C and 5% CO_2_. The Towne strain of HCMV was obtained from the ATCC (# VR 977), propagated under standard procedures and used at a multiplicity of infection (MOI) of 10 in all experiments. The recombinant Ad strains dL70-3, used as a control virus, and NGUS24i, encoding the I-*Sce*I enzyme, (both kind gifts of Frank Graham and Philip Ng, McMaster University, Hamilton, ON, Canada [15,16]) were grown and titrated in replication-permissive human 293A cells. The MOI of Ad viruses used was established separately depending on experimental procedure (see above for particular details).

### 3.2. Transfections

Lipofectamine reagent (Invitrogen/Life Technologies, Grand Island, NY, USA) was used according to manufacturer’s instructions to introduce pDRGFP [13,14] into T98Gs. Following transfection, cells were grown in non-selective medium for 48 h. Subsequently, puromycin (1 mg/mL) was added to the media to select for integrated pDRGFP. Cloning rings were used to isolate puromycin-resistant colonies. Colonies were separated and subjected to another cycle of puromycin selection prior to freezing and storage in liquid nitrogen. All colonies were tested to ensure the absence of GFP^+^ without DSB induction. Three stable clones of T98GpDRGFP were tested and produced comparable results in the assays described above. T98GpDRGFP clone 10 (Clone 10) was used for all experiments reported here.

### 3.3. Nucleofections

Nucleofection of T98Gs was optimized using a protocol provided by Amaxa Biosystems (program O-016). Each nucleofection used ~5 × 10^5^ cells. IE72 transient expression studies used either 6 µg of control vector pSG5 (Stratagene/Agilent Technologies, Santa Clara, CA, USA) or 6 µg of pSGIE72 [28]. p53 transient expression experiments used the following constructs in the listed quantities: 6 µg of vector pCDNA3-T7 alone (with the CMV promoter removed, as a control) or 6 µg of p53 derivative vectors containing (1) a wt p53 promoter-wt p53-green fluorescent protein (GFP) cassette (wtp53pCDNA3-T7) or (2) an equivalent vector cassette containing one of the following p53 mutations: K382R, R273H, R175H, R248W, G154V or an *N*-terminal multi-site phosphorylation mutant [29]. All of these have been previously described [22]. After nucleofection, cells were immediately transferred to plates containing coverslips and pre-warmed medium. 

### 3.4. Molecular Cloning

The pCDNA3-T7 vector was modified by insertion into its polylinker of a BamHI-NotI fragment containing DsRed2 (from pDsRed2-N1vector; Clontech Laboratories, Mountain View, CA, USA) to produce pCDNA3-DsRed. The wt p53 promoter-wt p53 coding sequence was cleaved from pCLNCX-p53pro-p53GFP [22] at the KpnI and AgeI sites. Finally, this fragment was inserted upstream of DsRed2 in the polylinker of pCDNA3-DsRed. This ultimately produced the pCDNA3-p53pro-p53DsRed construct. Dual transient expression studies with wt p53 and IE72 used 3 µg each of pCDNA3-p53pro-p53DsRed and pSGIE72. Alternatively, one of these constructs (3 µg) was transfected along with the corresponding control backbone of the other construct (3 µg).

### 3.5. DSB Repair Assay

The DSB repair assay was carried out as described previously [12]. The results reported were carried out in mock- or HCMV-infected Clone 10. 

### 3.6. Virus Infection

Clone 10 was synchronized in G_o_ by serum starvation for 3 d. Cells were then washed with PBS, trypsinized and re-plated at a density of 5 or 10 × 10^5^ cells/60-mm dish containing glass coverslips. After allowing 2 h for attachment, the cells were mock- or HCMV-infected. Twenty-four hours later the virus or mock inoculums were removed (unless otherwise noted). After an additional 24 h, cells were washed once with PBS and subsequently superinfected for 30 minutes in pre-warmed media. Super-infection was with control Ad dL70-3 or with I-*Sce*I-expressing Ad NGUS24i [12]. Cells were then re-fed with complete media. Cells were fixed and permeabilized 72 h later unless otherwise noted. Coverslips were scored for GFP^+^ cells and IE72^+^ cells.

### 3.7. Antibodies (Ab)

Primary mouse monoclonal antibodies (mAbs) used were: anti-pan actin (IgG_1_) (Neomarkers/Thermo Scientific, Fremont, CA, USA); anti‑hemagglutinin (HA)(12CA5) (IgG_2b_)(Abcam, Cambridge, MA, USA); anti-IE1 (IgG_2a_) (a kind gift from Bill Britt, University of Alabama, Birmingham, AL, USA); and anti-IE1 and -IE2 (Ch16.0; IgG_1_) (Virusys Corporation, Taneytown, MD, USA). Secondary Abs used were as follows: for immunoblot detection, horseradish peroxidase (HRP)-linked sheep anti-mouse (GE Healthcare Life Sciences, Pittsburgh, PA, USA), and for IF analysis, tetramethyl rhodamine isothiocyanate (TRITC)-conjugated anti-mouse IgG_1_, IgG_2a_ and IgG_2b_ (Jackson ImmunoResearch Laboratories, West Grove, PA, USA). 

### 3.8. Immunofluorescence (IF)

Cells were seeded into dishes containing glass coverslips. Coverslips were collected and processed at indicated times pi, as described previously [30]. 300–500 cells per coverslip were counted and scored for GFP^+^ and either HA^+^ or IE72^+^ cells in each experiment. All experimental results represent the average of at least two independent experiments. Error bars represent +/− one standard deviation (SD). Statistical analysis was performed using unpaired, two-tailed student t-tests. 

### 3.9. Immunoblotting

Virus- and mock-infected cells were harvested at 48, 72, and 96 hpi and lysates were processed as previously described [22,31]. 

### 3.10. GST Binding Studies

Overnight bacterial cultures (100 mL in LB + 100 μg/mL ampicillin) containing the GST fusion plasmids pGEX-human p53 (1-393) (Addgene plasmid #24860; [32]), pGEX72 [28] or pGEX-KG [33] were diluted 1:10 (to a final volume of 1 L LB/amp) and incubated an additional 1 h at 37 °C with shaking. Cultures were chilled to room temperature (RT), then 1 mL 0.5M IPTG was added. After an additional incubation at RT for 2 h, bacteria were pelleted at 5000 rpm for 10 min and cells were resuspended in 50 mL NETN + protease inhibitors (PIs) (20 mM Tris pH 8, 100 mM NaCl, 1 mM EDTA, 0.5% NP40, 2 mg/mL aprotinin, 2 mg/mL leupeptin, 100 mM DTT). This suspension was then frozen at −80 °C in 5 mL aliquots for future use. 

Assays were performed using one of the 5 mL aliquots thawed on ice. Fifty μL of a 10 mg/mL lysozyme solution was added and the cell suspension was incubated on ice for 30 min. The suspension was sonically disrupted to lyse the cells. Debris was pelleted at 10,000 rpm for 5 min and the supernatant transferred to a new tube. Sixty μL of a 50% glutathione agarose bead slurry (vol/vol in NETN) was added and rocked at 4 °C for 1 h. Beads were then washed in 500 μL NETN + PIs three times prior to final re-suspension in 500 μL NETN + PIs. Fifty μL of this bead suspension (25 μL bead equivalents) were incubated with 20 μL Laemmli reducing sample buffer (2% SDS, 10% glycerol, 100 mM DTT, 60 mM Tris pH 6.8, Bromophenol blue dye, aprotinin and leupeptin (2 μg/mL each)), and then boiled for 5 min. The resulting supernatant was loaded onto a 12% SDS-PAGE gel. The gel was stained with Coomassie dye to visualize the GST fusion proteins and to determine equivalent protein amounts for use in the following binding reactions. Roughly equivalent amounts of protein, as estimated from the Coomassie staining, were used in the reactions (approximately 30 μL beads of pGEX72 and pGEX-p53 and 7.5 μL of pGEX-KG were used in the reactions shown in Figure 4).

Pet24d-Rad51 [34] was *in vitro* translated using the Promega quick-coupled TNT reaction kit per manufacturer’s instructions. In each binding assay, five μL of translated Rad51 (or just TNT reaction mixture) was incubated while rocking for 1 h at 4 °C with the GST fusion proteins in a 500 μL reaction of NETN + PIs. After incubation, 40 μL of a 50% Protein A Sepharose slurry (vol/vol in NETN) was added to each tube (to increase the bead volume). Ten percent of the total volume (54 μL) was removed to serve as the input fraction (no washes of these beads followed). The beads in the remaining sample were pelleted and then washed four times with 500 μL of buffer (the first two washes in NETN, the last two in RIPA (150 mM NaCl, 1% Nonidet P-40, 0.5% deoxycholate, 0.1% SDS, 50 mM Tris (pH 8), 5 mM EDTA). After the final spin, 15 μL of urea sample buffer was added to the beads and the samples were boiled for 5 min. The supernatants were run on a 12% SDS‑PAGE gel. Gels were dried and exposed to Kodak X-omat film. 

## 4. Conclusions

A cell’s ability to repair insult to its DNA is a process that is essential to its survival. The concept that virus infection may alter this capability has begun to be investigated. In theory a virus might benefit from manipulation of the repair processes, while simultaneously (and likely corollary) degrading the integrity of a host’s genome. Existing studies have examined a variety of different repair pathways in the context of expression of a single viral protein and have assessed that protein’s capability to influence the repair of exogenously introduced damage in the cellular DNA [35,36,37,38,39,40,41,42,43,44,45,46]. The effects on repair during a complete infection have also been examined [12,47,48,49,50,51,52,53,54,55,56]. A handful of these studies have looked specifically at HDR [12,35,37,45,49]. The very large majority of these studies found viral protein expression (or full infection) decreased cellular repair capabilities.

Two cellular proteins, p53 and Rad51, are exceedingly important in the context of HDR. It is widely accepted that wt p53 regulates HDR, primarily through interactions with Rad51 (as reviewed in [21]). *In vitro* reactions have found p53 binds Rad51 and, by means of this interaction, inhibits strand exchange and branch migration of recombination intermediates [57]. An intact core DNA binding domain of the p53 protein is required for direct binding between the p53 and Rad51 proteins (and inhibition of the latter’s activities) [23,24,25]. It has also been shown that following induction of damage wt p53 inhibits transactivation of the Rad51 promoter, thereby acting as a repressor of Rad51 transcription and protein expression [23]. It has also been shown that a phosphorylatable N-terminus is important for regulation and binding of Rad51 [58]. Many tumors (and cell lines derived from them) have so called “hotspot” mutations in p53, including the T98G cells used in this study [20]. Reintroduction of wt p53 into these tumor cells reduced HDR via decreasing Rad51 activity [59].

Multiple viruses have been shown to interact with the Rad51 protein. Epstein-Barr Virus (EBV), a herpesvirus, and SV40 have both been found to require the presence of Rad51 within their viral replication centers. Knockdown of Rad51 in infected cells dramatically decreased viral replication [60,61]. The opposite was true for HIV; stimulation of Rad51 activity inhibited HIV replication, primarily due to an inhibitory effect of Rad51 on the virus’ Integrase enzyme [62]. 

The context of the T98G cellular environment is important for consideration of HDR in these cells. Under normal stress T98Gs can complete HDR and have normal levels of Rad51 [20]. Baseline levels of HDR in T98G cells after insult are quite high, perhaps in part due to the large proportion of these rapidly dividing cells being in the S/G2 phases of the cell cycle, during which HDR is most common [25,59]. However, the higher rate of HDR may also be attributable to the presence of the mutation in the p53 DNA binding domain (R273H) within these cells [20], which prevents p53’s normal binding to, and regulation of, Rad51.

The model in Figure 5 is proposed to explain the dichotomy of results between the T98G results reported here and our earlier HFF study. In wt cells p53 normally inhibits Rad51 activity. In the model, the prediction is that the mutant p53-containing T98G cells, where p53 cannot bind Rad51 [23,24,25], would have high baseline recombination rates, as seen. The model further predicts that, introduction on their own (indicated by the arrow in the figure) of either wt p53 or IE72 (which we have shown can bind Rad51 *in vitro*) into these cells would decrease HDR by their binding to and inhibiting Rad51. Co-expression of both wt p53 and IE72 did not inhibit Rad51. We propose in these experiments wt p53 and IE72 bound to one another, inhibiting normal p53 activity [27]. This seems a reasonable assumption, given previous reports that IE72 binds directly to p53 and inhibits core domain binding ability [27]. If the introduced wt p53 and IE72 were interacting and p53’s normal activity was inhibited, Rad51 would again be free to cause the high recombination rates observed.

Also in our model, in wt cells p53 normally inhibits Rad51, establishing baseline HDR activity levels. HCMV-infection of wt fibroblast cells causes p53 to become tightly associated with the viral replication centers [63]. We believe in an infected cell this viral manipulation of p53 prevents the normal binding of p53 and Rad51. The disruption of this normal condition in turn frees Rad51 to increase recombination in both the viral and cellular DNA. Experiments in wt cells found that expression of the single HCMV protein IE72 was sufficient to increase HDR levels [12]. The proposed model attributes the increase in HDR in wt cells to IE72 binding to wt p53, thereby inhibiting the ability of wt p53 to bind and regulate Rad51. 

The proposed model offers a mechanistic explanation for our previously reported results in wt HFFs [12]. The focus of the earlier HFF study was the recognition of viral manipulation of a cellular repair mechanism to its own ends in the context of a fully permissive infection. The experiments performed in T98G cells were expected to corroborate that data. Fortuitously, the particular microenvironment of the mutant glioblastoma cells allowed elucidation of what we believe to be the protein interactions responsible.

The results of this study may have implications for the initiation of glioblastomas. It has been reported that in excess of 90% of glioblastomas contain HCMV genetic material [64,65]. A latently HCMV-infected neural progenitor/stem cell which reactivated could, and most likely would, express IE72. We have found that expression of IE72 in a wt cell was capable of increasing unscheduled recombination, a hallmark of genetic instability and perhaps the first step toward hyperproliferation and gliomagenesis. The common perception of HCMV as a relatively harmless pathogen belie not only its capacity to cause serious birth defects, inflict direct DNA damage and downregulate repair of the cellular genome, but perhaps its capability to initiate an often lethal and devastating cancer. We have experiments underway to determine if the protein interactions found in this study are capable of intitiating oncogenic effects in tissue culture experiments.
